# Dosimetric Comparison of Helical Tomotherapy, Volume-Modulated Arc Therapy, and Fixed-Field Intensity-Modulated Radiation Therapy in Locally Advanced Nasopharyngeal Carcinoma

**DOI:** 10.3389/fonc.2021.764946

**Published:** 2021-11-05

**Authors:** Shan Lu, Huiqi Fan, Xueyuan Hu, Xin Li, Yingying Kuang, Deyang Yu, Shanshan Yang

**Affiliations:** ^1^ Department of Head and Neck Radiotherapy, Harbin Medical University Cancer Hospital, Harbin, China; ^2^ Department of Radiation Physics, Harbin Medical University Cancer Hospital, Harbin, China; ^3^ Department of Gynecological Radiotherapy, Harbin Medical University Cancer Hospital, Harbin, China

**Keywords:** radiotherapy technique, helical tomotherapy (HT), volume-modulated arc therapy (VMAT), fixed-field intensity-modulated radiation therapy (FF-IMRT), nasopharyngeal carcinoma (NPC)

## Abstract

**Objective:**

To compare the dosimetric parameters of different radiotherapy plans [helical tomotherapy (HT), volume-modulated arc therapy (VMAT), and fixed-field intensity-modulated radiation therapy (FF-IMRT)] for locally advanced nasopharyngeal carcinoma (NPC).

**Methods:**

A total of 15 patients with locally advanced NPC were chosen for this retrospective analysis and replanned for HT, VMAT, and FF-IMRT. The prescribed planning target volume (PTV) dose for the primary tumor and metastatic lymph nodes was 70 Gy (2.12 Gy/fraction, delivered over 33 fractions). The prescribed PTV dose for the high-risk subclinical region was 59.4 Gy (1.8 Gy/fraction, delivered over 33 fractions). The dosimetric parameters of the PTV and organs at risk (OARs) and the efficiency of radiation delivery were assessed and compared using the paired-samples t-test.

**Results:**

Compared with VMAT and FF-IMRT plans, HT plans significantly improved the mean conformity index (CI) and homogeneity index (HI). The HT plans reduced the maximum doses delivered to OARs, such as the brainstem, spinal cord, and optic nerves, and significantly reduced the volume delivered to the high-dose region, especially when examining the *V*_30_ value of the parotid glands. However, VMAT reduced the treatment time and improved the efficiency of radiation delivery compared with HT.

**Conclusions:**

For locally advanced NPC, the results showed that HT and VMAT possessed better target homogeneity and conformity, reducing the dose delivered to OARs compared with conventional FF-IMRT, with HT achieving the best effect. Among the techniques studied, VMAT had the shortest radiation delivery time. The results of this study can provide guidance for the selection of appropriate radiation technologies used to treat patients with locally advanced NPC who are undergoing concurrent chemoradiotherapy.

## 1 Introduction

Nasopharyngeal carcinoma (NPC) is among the most common head and neck cancer. The incidence of NPC has unique geographical and ethnic distribution patterns, with a high incidence in Asia, particularly in Southeast Asia. According to data from GLOBOCAN 2012, high incidence rates have been identified in several provinces of southeast China (such as Guangdong, Hongkong), Thailand, and the Philippines (1). NPC cases in China represented 48.62% and 50.34% of the incidence and mortality for all cases of Asia in 2012, respectively (2). Approximately 68% of NPC patients suffer from locally advanced disease at the time of diagnosis (3). Due to the complex anatomy and small region for surgery, the primary treatment modality for NPC is radiotherapy. Early-stage NPC can only be treated with radiotherapy. Locally advanced NPC is typically treated with concurrent chemotherapy and radiotherapy (CCRT) (4). However, CCRT can lead to considerable acute and late toxicities in many of the normal structures surrounding the nasopharynx, such as the pharyngeal mucosa, parotid glands, and cranial nerves (5, 6). Therefore, an increasing number of studies have begun to focus on reducing treatment-related side effects in patients with NPC undergoing CCRT.

Modern radiation techniques have evolved alongside the development of radiation equipment and advancements in radiation physics in recent years. Following the conventional three-dimensional conformal radiation therapy (3D-CRT) technique, intensity-modulated radiation therapy (IMRT) can achieve specific dosimetric and clinical objectives through a computer-aided optimization process (7), providing highly conformal dose distributions to the planning target volume (PTV), minimize the dose delivered to organs at risk (OARs) (8, 9), and significantly reduce acute and late toxicity (10, 11). Volume-modulated arc therapy (VMAT) and helical tomotherapy (HT) are gaining increasing attention compared with conventional fixed-field IMRT (FF-IMRT, 5/7/9-field). VMAT uses low monitor units (MUs) and treatment times, varying dose rates, and a dynamic multileaf collimator (MLC) based on a variable-speed rotational treatment paradigm. HT is a new computed tomography (CT)-based rotational IMRT that delivers a highly conformal dose distribution and spares OARs through the use of 51 independent beam directions and 64 pneumatically driven MLC leaves.

However, the high costs of primary equipment and maintenance for HT treatment systems result in increased treatment costs, which limits the use of this modality in clinical practice, especially in lower-income countries. This study aimed to assess three modern IMRT techniques (HT, VMAT, and FF-IMRT) in terms of the dosimetric parameters measured for the PTV and OARs in locally advanced NPC and to determine whether HT has significant dosimetric impacts that might justify the costs associated with this modality.

## 2 Materials and Methods

### 2.1 Patient Characteristics and CT Simulation

A total of 15 patients with Stage III/IVA NPC treated between February 2019 and February 2020 in our hospital were chosen for this research. All patients were staged according to the American Joint Committee on Cancer (AJCC) Manual for Staging of Cancer, 8th edition (12). The selection criterion was biopsy-proven squamous cell carcinoma. The ages of all eligible patients ranged from 39 to 68 years, and the mean and median ages were 56.7 and 60 years, respectively. A total of 10 patients received CCRT, 2 patients received radiotherapy and concurrent weekly targeted therapy with nimotuzumab, and 3 patients received both chemotherapy and targeted therapy with nimotuzumab during radiotherapy. The information for all patients is shown in **Table 1**. Thermoplastic head, neck, and shoulder masks were used to immobilize all patients in a supine position to perform CT simulations with 3-mm slice thickness using a Philips 16-slice Brilliance big bore CT scanner (Philips Medical Systems, Amsterdam, Netherlands) following the administration of intravenous contrast. Scanned images were obtained from the top of the head to the carina for all patients.

**Table 1:** Clinicopathological characteristics of the patients with nasopharyngeal carcinoma (NPC).

Characteristics	No. of Patients (*N* = 15)
Age(years)	
≤60	9
>60	6
Sex	
Male	13
Female	2
Pathology (SCC*)	
Poorly differentiated	10
Non-keratinizing	5
Clinical stage	
III	10
IVA	5
Concurrent therapy	
Chemotherapy	10
Targeted therapy	2
Chemotherapy + targeted therapy	3

*SCC, squamous cell carcinoma.

### 2.2 Target and Normal Tissue Volume Definition

All CT images were transferred to the Monaco 5.11 (Elekta AB, Stockholm, Sweden) planning system for contouring. For consistency, all contouring of the target and OARs was performed by the same radiation oncologists who specialized in head and neck radiotherapy. The target volume delineation of the NPC was based on the Radiation Therapy Oncology Group (RTOG) 2009 guidelines (13). The gross tumor volume (GTV_70_) was defined as the known gross disease of the nasopharynx. Grossly positive lymph nodes (GTV_nd_) were defined as any lymph node >1 cm or nodes with necrotic cancer. The clinical target volume for 59.4 Gy (CTV_59.4_) was defined as the region at high risk for microscopic disease, which included all potential routes of spread for primary and nodal diseases. The primary high-risk regions included the entire nasopharynx, anterior one-third of the clivus, skull base, pterygoid fossa, parapharyngeal space, inferior sphenoid sinus, posterior one-fourth of the nasal cavity/maxillary sinuses, inferior soft palate, and retrostyloid space. The common high-risk lymph node regions typically included the bilateral upper deep jugular, retropharyngeal area, and levels II, III, IV, and V lymph nodes. Level IB lymph nodes can be spared in selected patients. The OARs for NPC include the brainstem, spinal cord, optic nerves, optic chiasm, eyes, lens, temporal lobe, parotid glands, pituitary, temporomandibular joints (TMJ), mandible, oral cavity, brachial plexus, esophagus, and larynx. The PTV was defined as the CTV area + 3 mm.

### 2.3 Treatment Planning and Prescribed Doses

All treatment planning procedures were developed by the same radiation physicist to ensure consistency. The FF-IMRT and VMAT plans were designed using the Monaco planning system version 5.11, and the HT plans were designed in the tomotherapy planning system (Accuray Inc., Madison, USA). The FF-IMRT and VMAT plans were designed to be executed using the Elekta Synergy (Elekta Ltd., Crawley, UK), equipped with 8-MV photon beams, and the MLCi2 (40 pairs of MLC leaves, each with a 1-cm width at the isocenter). The prescribed PTV dose for the primary tumor (PTV_70_) and metastatic lymph nodes (PTVnd) was 70 Gy (2.12 Gy/fraction, delivered over 33 fractions). The prescribed PTV dose for the high-risk subclinical region was 59.4 Gy (1.8 Gy/fraction, delivered over 33 fractions). The details regarding the dose constraints for normal tissues within the NPC plans are summarized in **Table 2**.

**Table 2:** The dose–volume constraints of normal tissues in NPC.

Structures	Dose–volume constraints
Brainstem	*D_max_* < 54 Gy
Spinal cord	*D_max_* < 45 Gy
Optic nerves	*D_max_* < 54 Gy or D1 < 60Gy
Optic chiasm	*D_max_* < 54 Gy
Lens	*D_max_* < 8 Gy
Eyes	*D_max_* < 40 Gy
Pituitary	*D_max_* < 60 Gy
Mandible	*D_max_* < 70 Gy
TMJ	*D_max_* < 70 Gy
Brachial plexus	*D_max_* < 66 Gy
Oral cavity	V40 < 40%
Parotid gland	V30 < 50%
Temporal lobes	*D_max_* < 60 Gy or D1 < 65Gy
Larynx	V45 < 40%
Esophagus	V45 < 40%

#### 2.3.1 HT Plans

The HT plans were generated using a tomotherapy planning station with a 6-MV X-ray and performed on the Tomo HD (Accuray Inc., Madison, USA). The parameters for beamlet calculation included a field width of 2.5 cm, a pitch value of 0.287, a modulation factor of 3, and a normal dose calculation grid.

#### 2.3.2 VMAT Plans

The VMAT plans were generated in the Monaco 5.11 planning system, and an 8-MV X-ray in a Synergy linear accelerator was used. The VMAT plans were designed using a beam with double 360° arcs, featuring 100 control points per arc. All VMAT plans were designed using the Monte Carlo algorithm in the Monaco 5.11 planning system.

#### 2.3.3 FF-IMRT Plans

The FF-IMRT plans were generated in the Monaco 5.11 planning system, and an 8-MV X-ray in a Synergy linear accelerator was used. Nine evenly distributed coplanar fields with gantry angles of 200°, 240°, 280°, 320°, 0°, 40°, 80°, 120°, and 160° were used, featuring 20 control points in each beam. All FF-IMRT plans were prepared using the Monte Carlo algorithm in the Monaco 5.11 planning system. The optimization functions of the FF-IMRT plans were the same as those in the VMAT plans. The dynamic MLC (DMLC, sliding window) technique was used in the FF-IMRT plans.

### 2.4 Plan Evaluation Parameters

The data obtained in the dose–volume histogram (DVH) for all plans were analyzed, and plan comparisons focused on the following parameters.

#### 2.4.1 PTV Coverage

The dose that received 98% volume of the PTV (*D_98%_*), the dose that received 50% volume of the PTV(*D_50%_*), the dose received 2% volume of the PTV(*D_2%_*), the mean dose (*D_mean_*), the conformity index (CI), and the homogeneity index (HI) were quantified to evaluate PTV coverage. The CI was used to evaluate the conformity of the prescribed dose distribution:


$$CI=\frac{V_{t,ref}}{V_{t}}\times \frac{V_{t,ref}}{V_{ref}}$$

where *V_t,ref_*, *V_t_*, and *V_ref_* denote the target volume that received the prescribed dose, the target volume, and the total volume covered by the prescribed dose, respectively. The CI ranges from 0 to 1, with a high CI indicating a high conformal dose delivery to the target. In accordance with The International Commission on Radiation Units and Measurements (ICRU) report No. 83 (14), the HI was calculated using the following formula:


$$HI=\frac{D_{2\%}-D_{98\%}}{D_{50\%}}$$

HI was used to evaluate the homogeneity of the dose distribution. The low HI value indicates good homogeneity of the target volume.

#### 2.4.2 Organs at Risk

For patients with NPC, the following values were determined: the maximum doses (*D_max_*) delivered to the brainstem, spinal cord, optic nerves, optic chiasm, lens, eyes, pituitary, mandible, TMJ, and brachial plexus; the mean doses (*D_mean_*) delivered to the larynx, oral cavity, and esophagus; the volume that received 30 Gy (*V*_30_) and the *D_mean_* for the parotid glands; the dose delivered to 1% of the OAR volume (*D*_1_); and the maximum dose (*D_max_*) delivered to the temporal lobes.

#### 2.4.3 Treatment Time

The treatment delivery time for each plan was determined and compared.

#### 2.4.4 Data Analysis

All plans were normalized to deliver the prescribed dose to 95% volume of the PTV to allow for comparison across results. The data collected from the DVHs for the PTV and OARs were analyzed using SPSS 19.0 (SPSS, Inc., Chicago, IL, USA). Significant differences were tested using the paired-samples t-test. A *p* < 0.05 was considered significant.

## 3 Results

### 3.1 PTV Coverage

The mean PTV_70_, PTV_nd_, and PTV_59.4_ values for NPC were 53.6 ± 30.3 cc (11.3–106.5 cc), 41.6 ± 38.9 cc (6.9–107.9 cc), and 674.0 ± 142.4 cc (503.3–874.0 cc), respectively. All HT, VMAT, and FF-IMRT plans were normalized to cover 95% of the PTV with ≥100% of the prescribed dose. The *D_max_* constrained in the PTV was <110% of the prescription dose.

**Figure 1:**
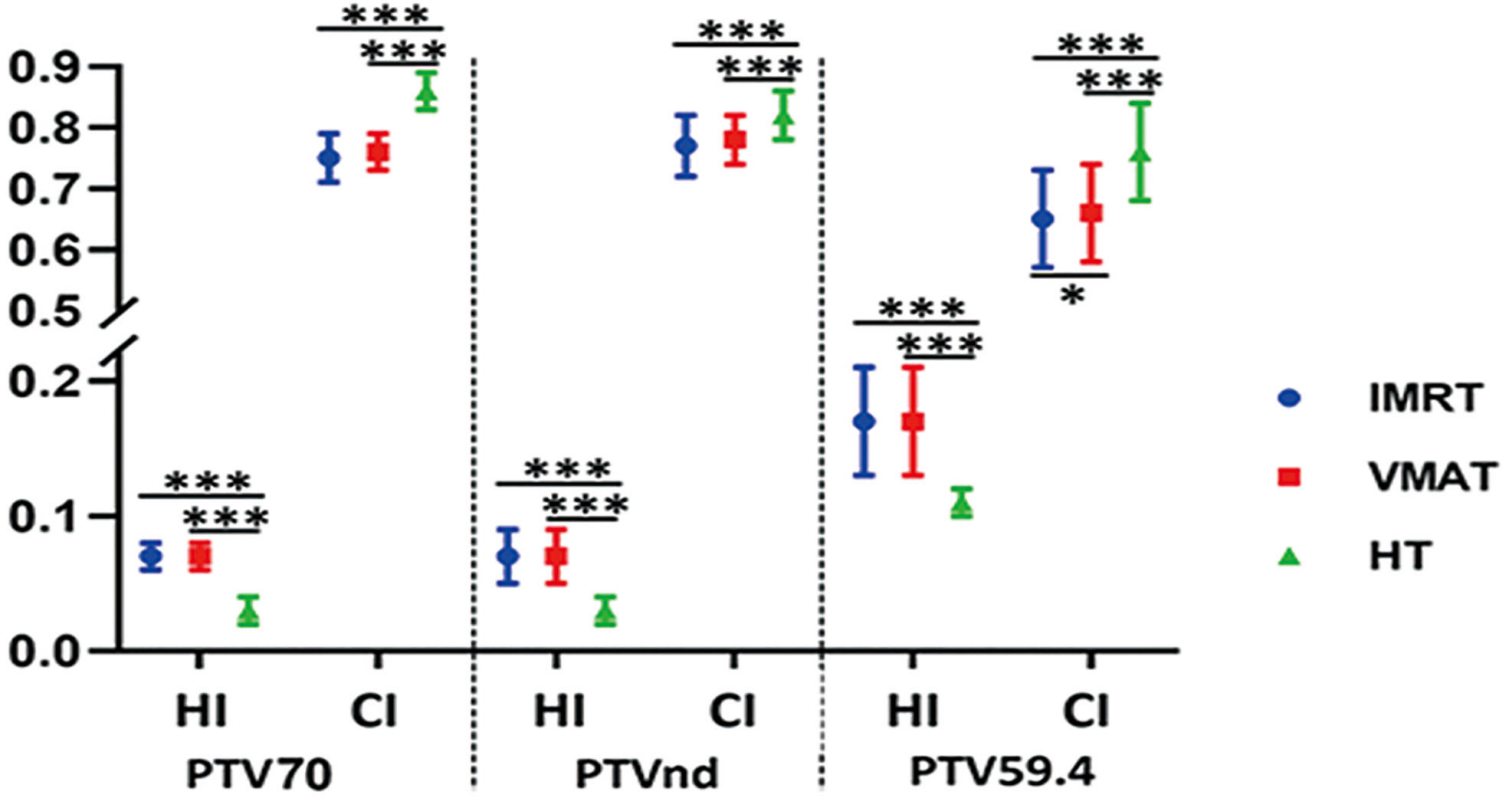
Conformity index (CI) and homogeneity index (HI) for planning target volume (PTV) with intensity-modulated radiation therapy (IMRT; circle), volume-modulated arc therapy (VMAT; square), and helical tomography (HT; triangle). *p < 0.05, **p < 0.01, ***p < 0.001.

**Figure 2:**
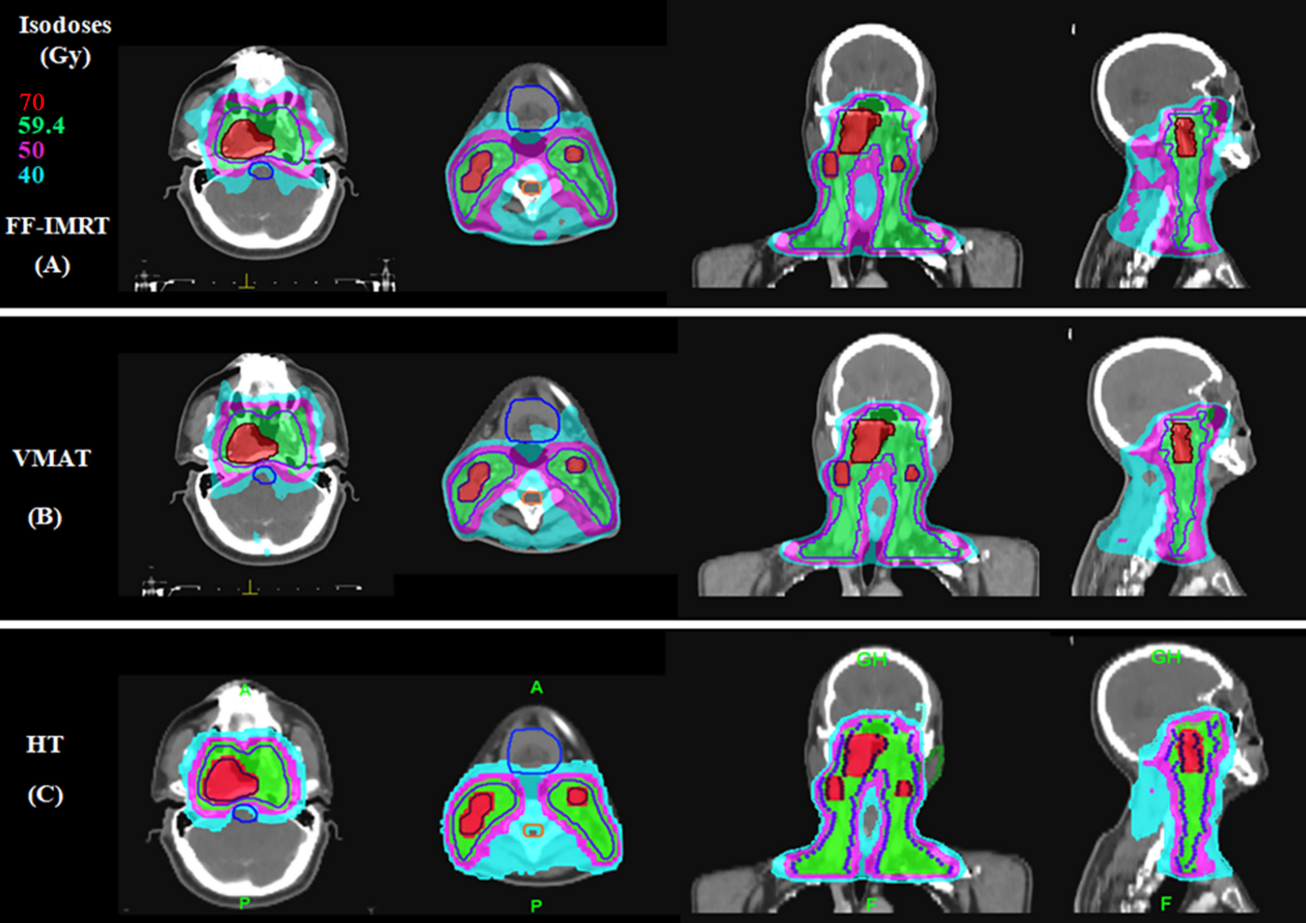
Typical dose distributions for the three plans in locally advanced nasopharyngeal carcinoma (NPC). **(A)** Fixed-field intensity-modulated radiation therapy (FF-IMRT), **(B)** volume-modulated arc therapy (VMAT), and **(C)** helical tomography (HT) plans.

**Figure 3:**
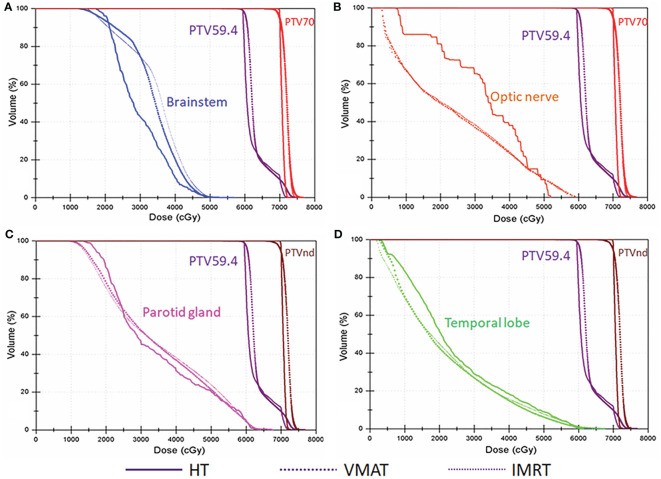
Typical dose–volume histograms for the three plans in locally advanced nasopharyngeal carcinoma (NPC). Dose–volume histograms for planning target volume of 70 Gy (PTV_70_; red), planning target volume for metastatic lymph nodes (PTV_nd_; brown), planning target volume of 59.4 Gy (PTV_59.4_; purple), **(A)** the brainstem (blue), **(B)** optic nerve (orange), **(C)** parotid gland (pink), and **(D)** temporal lobe (green).

The detailed results are shown in **Table 3**. The conformal and homogeneous dose distribution to the PTV target for the HT plans, as assessed using the CI and HI, respectively, were significantly better than those for the VMAT and FF-IMRT plans (*p* < 0.001; **Figure 1**). The HT plans also had the best *D_mean_* value (*p* < 0.001), approaching the prescription dose, demonstrating significant advantages over the other two plans. Compared with the conventional FF-IMRT plans, the VMAT plans did not show significant superiority for HI and CI (*p >* 0.05), and only the CI of PTV_59.4_ was better for VMAT compared with FF- IMRT (*p* = 0.016). Typical dose distributions and dose–volume histograms for the three plans are presented in **Figures 2** and **3**.

**Table 3:** Dosimetric parameters for PTV of three plans.

Parameters	IMRT	VMAT	HT	p*		
				VMAT *vs*. IMRT	HT *vs*. IMRT	HT *vs*. VMAT
PTV70						
*D_mean_* (Gy)	72.10 ± 0.49	72.24 ± 0.37	70.63 ± 0.23	0.117	<0.001	<0.001
HI	0.07 ± 0.01	0.07 ± 0.01	0.03 ± 0.01	0.217	<0.001	<0.001
CI	0.75 ± 0.04	0.76 ± 0.03	0.82 ± 0.04	0.086	<0.001	<0.001
PTVnd						
*D_mean_* (Gy)	72.11 ± 0.52	72.30 ± 0.25	70.63 ± 0.25	0.409	<0.001	<0.001
HI	0.07 ± 0.02	0.07 ± 0.02	0.03 ± 0.01	0.726	<0.001	<0.001
CI	0.77 ± 0.05	0.78 ± 0.04	0.82 ± 0.04	0.184	<0.001	<0.001
PTV59.4						
*D_mean_* (Gy)	62.50 ± 0.60	62.47 ± 0.49	60.85 ± 0.43	0.765	<0.001	<0.001
HI	0.17 ± 0.04	0.17 ± 0.04	0.11 ± 0.01	0.082	<0.001	<0.001
CI	0.65 ± 0.08	0.66 ± 0.08	0.76 ± 0.08	0.016	<0.001	<0.001

*P value was computed by paired t test.

### 3.2 OARs

The DVH data for the OARs in NPC are listed in **Table 4**. Our results showed that the *D_max_* values for the brainstem, spinal cord, optic nerves, lens, eyes, pituitary, TMJ left, and temporal lobes assessed in HT plans were significantly lower than those in FF-IMRT and VMAT plans (*p* ≤ 0.01). HT also resulted in significantly improved dose sparing based on the *V*_30_ value of the parotid glands, the *D*_1_ of the temporal lobes, and the *D_mean_* of the larynx, compared with FF-IMRT and VMAT (*p* < 0.05). Compared with FF-IMRT, VMAT significantly decreased the *D_max_* of the brainstem, spinal cord, optic nerve, eyes, TMJs, and temporal lobe and decreased the *D_mean_* of the larynx and esophagus (*p* < 0.05).

**Table 4:** Dose–volume histogram comparisons for the main OARs of three plans.

OARs	IMRT	VMAT	HT	p*		
				VMAT *vs*. IMRT	HT *vs*. IMRT	HT *vs*. VMAT
Brainstem						
*D_max_* (Gy)	53.77 ± 1.33	53.16 ± 1.26	51.84 ± 1.95	0.006	<0.001	0.002
Spinal cord						
*D_max_* (Gy)	43.99 ± 1.03	42.99 ± 1.15	41.34 ± 1.57	0.022	<0.001	<0.001
Optic nerve left						
*D_max_* (Gy)	55.46 ± 2.98	54.46 ± 4.13	52.07 ± 3.14	0.040	<0.001	0.002
Optic nerve right						
*D_max_* (Gy)	55.05 ± 2.39	55.11 ± 3.13	50.90 ± 3.24	0.881	<0.001	<0.001
Optic chiasm						
*D_max_* (Gy)	42.52 ± 11.57	41.88 ± 11.82	42.67 ± 7.26	0.378	0.912	0.600
Lens left						
*D_max_* (Gy)	7.43 ± 1.64	7.16 ± 1.94	5.47 ± 0.82	0.346	<0.001	0.001
Lens right						
*D_max_* (Gy)	7.69 ± 1.28	7.46 ± 1.36	5.82 ± 0.62	0.502	<0.001	<0.001
Eye left						
*D_max_* (Gy)	35.72 ± 4.70	33.06 ± 6.68	28.13 ± 5.25	0.011	<0.001	0.001
Eye right						
*D_max_* (Gy)	35.92 ± 3.29	33.62 ± 5.62	26.62 ± 4.59	0.018	<0.001	<0.001
Pituitary						
*D_max_* (Gy)	58.22 ± 4.97	58.54 ± 5.35	52.32 ± 6.78	0.375	<0.001	<0.001
Mandible						
*D_max_* (Gy)	67.39 ± 3.87	67.76 ± 3.83	66.61 ± 4.20	0.249	0.234	0.095
TMJ left						
*D_max_* (Gy)	61.07 ± 2.53	59.16 ± 3.54	57.85 ± 3.42	0.001	<0.001	0.004
TMJ right						
*D_max_* (Gy)	60.23 ± 4.88	58.58 ± 5.17	57.10 ± 5.36	0.016	<0.001	0.027
Brachial plexus left						
*D_max_* (Gy)	63.93 ± 2.19	64.32 ± 2.18	63.25 ± 3.79	0.132	0.363	0.177
Brachial plexus right						
*D_max_* (Gy)	64.20 ± 2.63	64.79 ± 3.00	63.27 ± 4.10	0.074	0.171	0.022
Oral cavity						
*D_mean_* (Gy)	38.06 ± 1.48	38.44 ± 1.79	37.68 ± 1.57	0.404	0.356	0.229
Parotid gland left						
*D_mean_* (Gy)	33.81 ± 1.34	33.38 ± 1.87	33.64 ± 1.04	0.471	0.683	0.664
V30 (%)	50.58 ± 2.52	49.96 ± 2.70	45.23 ± 1.73	0.537	<0.001	<0.001
Parotid gland right						
*D_mean_* (Gy)	34.71 ± 1.08	33.86 ± 1.72	33.87 ± 1.13	0.036	0.048	0.975
V30 (%)	51.15 ± 1.68	50.44 ± 2.06	45.56 ± 2.14	0.359	<0.001	<0.001
Temporal lobe left						
*D_max_* (Gy)	65.16 ± 3.94	64.79 ± 3.80	62.09 ± 3.73	0.071	<0.001	<0.001
D1 (Gy)	59.38 ± 1.26	59.13 ± 1.84	58.23 ± 1.28	0.429	0.006	0.028
Temporal lobe right						
*D_max_* (Gy)	63.90 ± 1.39	64.08 ± 1.69	60.46 ± 0.77	0.659	<0.001	<0.001
D1 (Gy)	58.87 ± 0.85	59.35 ± 1.05	58.00 ± 1.00	0.080	0.001	<0.001
Larynx						
*D_mean_* (Gy)	43.22 ± 1.08	41.99 ± 2.07	39.56 ± 0.98	0.024	<0.001	0.001
Esophagus						
*D_mean_* (Gy)	30.41 ± 6.63	28.80 ± 6.58	28.18 ± 5.91	0.007	<0.001	0.371

*p value was computed by paired t test.

### 3.3 Treatment Time

The treatment delivery times for the three treatment techniques were determined to study the execution efficiency of the three radiotherapy technologies. The mean treatment delivery times for FF-IMRT, VMAT, and HT were 7.49 ± 0.32, 4.40 ± 0.29, and 7.59 ± 0.40 min, respectively. Compared with FF-IMRT and HT, VMAT had the highest execution efficiency.

## 4 Discussion

Cancer is a major public health problem worldwide and is expected to represent the leading cause of death in every country during the twenty-first century. Worldwide, 129,079 newly diagnosed NPC cases and 72,987 NPC-related deaths were reported in 2018 (15). In southeast China, the incidence of NPC is higher than in most other countries. Due to the unique anatomical structure of the nasopharynx and the high sensitivity of NPC to ionizing radiation, radiotherapy is the preferred treatment. With the continued development of radiotherapy technology, local NPC control and survival have significantly improved over the past half-century (16). CCRT is a standard treatment for locally advanced NPC. However, 15.8% of NPC patients experience recurrence within 5 years after radiotherapy, especially among patients with advanced disease (17), indicating that novel treatment approaches remain necessary.

Molecular targeted therapy and immunotherapy represent two new approaches to NPC. Epidermal growth factor receptor (EGFR) is highly expressed in NPC compared with other solid tumors (18). A retrospective analysis showed that an EGFR inhibitor (e.g., nimotuzumab) combined with CCRT was beneficial for treating locally advanced NPC (19). Immunotherapy has become a hotspot for cancer treatment research. Clinical trial data have shown that immune-checkpoint inhibitors, such as those against programmed cell death 1 (PD-1), can be effective in patients with recurrent or metastatic NPC (20, 21). However, the feasibility of concurrent immunotherapy and radiotherapy in locally advanced NPC remains unclear.

For patients with recurrent disease, the primary cause of local recurrence is insufficient irradiation dose delivered to the target area, which is limited by the dose tolerance of the surrounding OARs. Therefore, exploring feasible and optimized radiotherapeutic techniques is critical to achieving highly conformal treatment plans and acquiring good OAR sparing results. A number of constraining organs surround the irradiation area in NPC, and radiotherapy may cause acute or late adverse effects of these structures (e.g., acute mucositis, xerostomia, and temporal lobe neuropathy). Our study aimed to decrease the radiation doses and irradiated volumes of these structures.

CCRT has demonstrated improved survival benefits in locally advanced NPC, but it typically induces acute and late toxicities, sometimes emerging months or even years after treatment completion (22). A phase II study showed that the addition of an anti-EGFR antibody to radiotherapy enhanced radiotherapy-related acute toxicities to the skin and mucosa (23). Therefore, attention should be paid to side effects related to the treatment and their effects on quality of life among patients with NPC, in addition to the local tumor control rate. As one of the few hospitals in China equipped with several advanced linear accelerators (nine linear accelerators, including Versa HD and HT), our hospital had the unique capacity to perform a dosimetric study of IMRT, VMAT, and HT. Reports comparing the dosimetric parameters of FF-IMRT, VMAT, and HT with regard to the PTV and OARs in NPC are rare. Therefore, this study aimed to estimate which of the three radiotherapy techniques were dosimetrically superior to provide guidance regarding technique selection for patients with locally advanced NPC.

Due to the complex anatomy and various OARs closely positioned to NPC target tissues, radiotherapy for NPC is technically challenging and highly complex. Previous studies have confirmed that the modern IMRT is associated with a significantly steeper dose gradient surrounding the target region compared with conventional 3D-CRT (17, 24). Growing evidence suggests that HT can sculpt radiation doses to fit the complex shapes of tumorous regions, avoiding the delivery of high-dose radiation to OARs through the rapid opening and closing of leaves in a collimator that rotates around the patient (25). Therefore, HT is frequently used to treat various diseases (26–29), including NPC (30). In this study, the results showed that the three IMRT techniques met the clinical demands of NPC therapy but HT presented with a sharp dose gradient associated with optimal HI and CI values. Based on these results, HT is the recommended radiotherapy technique for ensuring local tumor control and improving patient prognosis when treating NPC with radiotherapy.

In addition to improved target conformity and homogeneity, HT demonstrated significantly better performance in sparing the surrounding OARs compared with the other techniques. The nasopharynx is adjacent to several critical organs, such as the brainstem, lens, and optic nerves. To protect critical organs, some parts of the tumor are often underdosed, which may lead to a low local control rate (31). The delivery of high doses of radiation to large volumes of normal tissues typically results in the development of severe adverse effects, such as dysphagia and radiation mucositis, which may interrupt radiation treatment. Therefore, decreasing the dose and volume delivered to normal tissues that surround the targeted radiation regions is crucial. In our study, the results showed that compared with the FF-IMRT and VMAT plans, the HT plans significantly decreased the *D_max_* of the brainstem, spinal cord, optic structures, pituitary, TMJ, temporal lobes, and larynx (**Table 4**). Moreover, the HT plans decreased the *V*_30_ value of the parotid glands compared with the FF-IMRT (*p* < 0.001) and VMAT (*p* < 0.001) plans. Therefore, HT plans may decrease radiotherapeutic adverse effects by reducing the doses and volumes of normal organ irradiation.

The significant advantages of HT plans over FF-IMRT and VMAT plans with regard to PTV coverage and OAR sparing are associated with the following features. First, the linear accelerator used during HT can rotate 360° continuously, with 51 optimized beam angles combined with a continuously moving couch. Second, HT delivers radiation in the form of a helical tomoscan by using constant beam widths of 1, 2.5, and 5 cm. Finally, HT is equipped with a pneumatic binary MLC system with rapid leaf transition times. In addition, the onboard megavoltage CT (MVCT) of HT allows daily setup validation. The margin expanding from the CTV to the PTV can be decreased because setup errors are reduced by daily setup verification, resulting in a reduced dose delivered to OARs. The MVCT can be used to perform adaptive radiotherapy planning, which can eliminate volume variations delivered to the target and OARs between intrafractions.

HT plans also have some drawbacks. Vernat and Pasquier reported that HT increases the normal tissue volume in the low-dose region compared with IMRT and VMAT when applied to oropharyngeal and prostate cancers (32, 33). Xie has reported that the HT plan increased the *V*_5_ and *V*_10_ values of the lung and heart compared with IMRT and VMAT plans for left-sided breast cancer (34). Therefore, the application of HT in lung and breast cancers remains controversial. In NPC cases, most OARs are serially organized structures, closely related to *D_max_*. Thus, our study focused on the *D_max_* of most OARs, except for the parotid glands, rather than examining the low-dose volumes. For the parotid glands, the *V*_30_ and *D_mean_* were evaluated according to RTOG guidelines. Our results showed that HT could significantly decrease the *V*_30_ value of the parotid glands.

Compared with FF-IMRT, VMAT exhibited better OAR sparing abilities. In addition, compared with FF-IMRT and HT, VMAT reduced the treatment time and improved the treatment efficiency while ensuring the treatment effect. Compared with FF-IMRT and HT, VMAT reduced the treatment delivery time by 41.3% and 42%, respectively. Several studies have reported that VMAT achieves higher PTV dose conformity and better OAR sparing abilities with a shorter treatment delivery time than FF-IMRT for various cancers (29, 35, 36). Shortened treatment times may reduce the influence of uncertain factors, including the probability that patients will move and suffer discomfort. Therefore, VMAT is the most appropriate treatment technique for patients who cannot remain in a stable position for long times due to physical or mental discomfort.

Our study had some limitations. First, we only used a nine-field coplanar arrangement for FF-IMRT and a two-arc coplanar beam configuration for VMAT to reduce the complexity of comparisons, as evidence suggests that these two techniques are the best options for obtaining better target coverage with enhanced sparing of the OARs for FF-IMRT and VMAT radiotherapy (37–39). Additionally, the limited sample size in our study may result in insufficient statistical power to identify significance for some of the dosimetric parameters. Therefore, further clinical trials with large sample sizes focusing on the clinical significance of HT in NPC are essential in the future.

## 5 Conclusion

For patients with locally advanced NPC, the HT and VMAT plans showed improvements in target coverage and OAR sparing compared with the FF-IMRT plans. The HT plans achieved optimal conformity and homogeneity for PTV coverage, with optimal OAR sparing. VMAT was associated with reduced treatment time and improved radiation delivery efficiency, which can reduce the patients’ discomfort and the probability of movement during treatment. In addition, the treatment costs of VMAT are lower than those of HT. Therefore, our results may provide guidance for technique selection in patients with locally advanced NPC who are undergoing CCRT.

## Data Availability Statement

The original contributions presented in the study are included in the article/supplementary material. Further inquiries can be directed to the corresponding authors.

## Ethics Statement

The studies involving human participants were reviewed and approved by the Ethics Committee of Harbin Medical University Cancer Hospital (Harbin, China) was obtained. The patients/participants provided their written informed consent to participate in this study. Written informed consent was obtained from the individual(s) for the publication of any potentially identifiable images or data included in this article.

## Author Contributions

DY and SY designed the study. SL and HF contoured the targets and OARs. DY, XH, and XL performed the treatment planning design. SL and DY wrote and revised the manuscript. DY and YK collected the data. DY and SY polished the language. All authors contributed to the article and approved the submitted version.

## Funding

This work was supported by grants from the Haiyan Research fund of Harbin Medical University Cancer Hospital (JJMS2021-27 and JJQN2021-03) and Project of Precise Radiotherapy Spark Program (2019-N-11-11).

## Conflict of Interest

The authors declare that the research was conducted in the absence of any commercial or financial relationships that could be construed as a potential conflict of interest.

## Publisher’s Note

All claims expressed in this article are solely those of the authors and do not necessarily represent those of their affiliated organizations, or those of the publisher, the editors and the reviewers. Any product that may be evaluated in this article, or claim that may be made by its manufacturer, is not guaranteed or endorsed by the publisher.

## Abbreviations

NPC, nasopharyngeal carcinoma; HT, helical tomotherapy; VMAT, volume-modulated arc therapy; FF-IMRT, fixed-field intensity-modulated radiation therapy; OARs, organs at risk; CI, conformity index; HI, homogeneity index; CCRT, concurrent chemotherapy and radiotherapy; PTV, planning target volume; MUs, monitor units; MLC, multileaf collimator; AJCC, American Joint Committee on Cancer; RTOG, Radiation Therapy Oncology Group; TMJ, temporomandibular joints; CTV, clinical target volume; DVH, dose–volume histogram; *D*_98%_, the dose that received 98% volume of the PTV; *D_max_*, the maximum dose; *D_mean_*, the mean dose; EGFR, epidermal growth factor receptor; PD-1, programmed cell death 1.
